# Common and Key Differential Pathogenic Pathways in Desminopathy and Titinopathy

**DOI:** 10.7150/ijms.97797

**Published:** 2024-08-01

**Authors:** Qiuxiang Li, Liqun Xu, Huiqian Duan, Huan Yang, Yue-Bei Luo

**Affiliations:** 1 Department of Neurology, Xiangya Hospital, Central South University, Changsha, China.; 2 Research Center for Neuroimmune and Neuromuscular disorders, Xiangya Hospital, Central South University, Changsha, China.; 3 Center for Molecular Medicine, Karolinska Institutet, Stockholm, Sweden.; 4 Division of Rheumatology, Department of Medicine Solna, Karolinska Institutet, Stockholm, Sweden.

**Keywords:** myofibrillar myopathy, desminopathy, titinopathy, proteomics, cytoskeleton, glycolysis

## Abstract

Myofibrillar myopathy (MFM) is a group of hereditary myopathies that mainly involves striated muscles. This study aimed to use tandem mass tag (TMT)-based proteomics to investigate the underlying pathomechanisms of two of the most common MFM subtypes, desminopathy and titinopathy. Muscles from 7 patients with desminopathy, 5 with titinopathy and 5 control individuals were included. Samples were labelled with TMT and then underwent high-resolution liquid chromatography-mass spectrometry analysis. Compared with control samples, there were 436 differentially abundant proteins (DAPs) in the desminopathy group and 269 in the titinopathy group. When comparing the desminopathy with the titinopathy group, there were 113 DAPs. In desminopathy, mitochondrial ATP production, muscle contraction, and cytoskeleton organization were significantly suppressed. Activated cellular components and pathways were mostly related to extracellular matrix (ECM). In titinopathy, mitochondrial-related pathways and the cellular component ECM were downregulated, while gluconeogenesis was activated. Direct comparison between desminopathy and titinopathy revealed hub genes that were all involved in glycolytic process. The disparity in glycolysis in the two MFM subtypes is likely due to fiber type switching. This study has revealed disorganization of cytoskeleton and mitochondrial dysfunction as the common pathophysiological processes in MFM, and glycolysis and ECM as the differential pathomechanism between desminopathy and titinopathy. This offers a future direction for targeted therapy for MFM.

## Introduction

Myofibrillar myopathy (MFM) is a group of hereditary muscular disorder with heterogeneous involvement of musculoskeletal system, heart and peripheral nerves. The pathology of MFM is characterized by sarcomere and Z disc disarray, mitochondrial malformation, and abnormal proteins aggregation within muscle fibers. At least 17 genes have been identified as disease-causing for MFM 1. In fact, MFM overlaps both phenotypically and genetically with other types of hereditary myopathies, such as limb-girdle muscular dystrophy and distal myopathy. The encoded proteins of these genes are mostly constitutive or functional members of the basic contractile unit of muscles, sarcomere or protein degradation machinery.

In our previously reported Chinese MFM cohort, *DES* and *TTN* are the most common pathogenic genes 2. Desminopathy patients often present with distal myopathy and arrhythmia in adulthood, while titinopathy patients more frequently exhibit joint deformity and respiratory insufficiency, with significant variation in the age of onset. The encoded proteins (desmin and titin) of both genes are associated with sarcomere. Titin spans half of a sarcomere and acts as a spring for muscle contraction. It also provides the molecular blueprint for sarcomere assembly and regulates the actin-myosin interaction by altering the ATPase activity of myosin 3, 4. The possible pathomechanisms of mutated titin include defective sarcomere assembly, autophagy and calcium handling machinery, and mitochondrial dysfunction 5, 6. Desmin 'wraps' around the Z disc and connects the neighbouring sarcomeres so that the parallel myofibrils can be functionally linked, as well as linked to other cellular organelles such as the mitochondria, sarcolemma, and nucleus. Mutations in *DES* can lead to thin filament destabilization, defective myogenesis and mitochondria, reduced cell elasticity, and force generation 7-11.

So far there are several proteomic studies on desminopathy, but very few have investigated the proteome of titinopathy patients or models 5, 12-16. Nor is there any study comparing the proteomic changes between the two diseases. The present study aimed to perform comparative proteomic analysis on desminopathy and titinopathy in order to identify the common and distinctive pathophysiology of these two MFM subtypes.

## Material and Methods

### Study objects

Muscle biopsies from 7 cases with desminopathy, 5 cases with titinopathy and 5 control individuals were included for this study. The diagnosis of desminopathy and titinopathy was confirmed by next generation sequencing for neuromuscular disorders. Control muscles were from biopsies of individuals who were excluded from any neuromuscular disorders based on clinical presentation and laboratory test results. Clinical and laboratory data of patients and controls were collected and presented in Supplementary Table 1. All recruited patients have signed consent forms. This study was approved by the Ethics Committee of Xiangya Hospital of Central South University (201603284).

### Sample preparation and TMT labeling

Muscle specimens of biceps brachii or quadriceps femoris of all patients were snap frozen by isopropene cooled in liquid nitrogen. Samples were ground and lysed with SDS buffer (Beyotime, China) containing 50 mM Tris, 1% SDS, sodium pyrophosphate, β-glycerophosphate, sodium orthovanadate, sodium fluoride and leupeptin. The lysate was sonicated using ultrasonic cell disruptor (Scientz, China). The mixture was centrifuged at 12,000 rpm for 10 min twice. Then supernatant was collected for quantification of protein concentration using the BCA method. From each sample, 50 μg of protein was diluted by 25 mM DTT (Adamas-beta, China) to make the latter 5mM. After incubating at 55 °C for 55 min and then returning to room temperature, the solution was diluted by iodoacetamide (BBI, China) to make the latter 10mM. Precooled acetone of six times the volume of the protein solution was added for precipitation. After incubation for 4 hr, the solution was centrifuged at 8000 g for 10 min at 4 °C and the precipitate was collected. After evaporation for 3 min, the precipitate was redissolved by 100 μL TEAB of 200 mM (Sigma-Aldrich, US; pH 8.5). Trypsin of 1mg/ml (protein: enzyme ratio= 50:1 (m/m)) was then added for digestion at 37 °C for 12 h. Finally, samples were lyophilized and stored at -80 °C.

For Tandem Mass tags (TMT, Thermo Fisher, US) labelling, the lyophilized samples were resuspended in 50 μL 100 mM TEAB. Acetonitrile of 20 μL (Thermo Fisher) were added at room temperature. The centrifuged reagents were dissolved for 5 min and mixed for centrifugation and repeat this step once. Each sample was labelled by 10 μL 18-plex TMT label reagent (A52047, Thermo Fisher) and incubated at room temperature for 1 hr. Finally, 5 µL of 5% hydroxylamine (Sigma-Aldrich) were added to each sample and incubated for 15 min to terminate reaction. The labelled peptide solution was lyophilized and stored at -80 °C.

### High-resolution liquid chromatography-mass spectrometry analysis

Reverse Phase separation was performed on an 1100 HPLC System (Agilent, US) using an Agilent Zorbax Extend RP column (5 μm, 150 mm × 2.1 mm). Mobile phases A and B (2% and 98% acetonitrile in HPLC water) were used for reverse-phase gradient. TMT labelled peptides were separated at a fluent flow rate of 300 μL/min applying the following gradient: 0 to 8 min, 98% A; 8 to 8.01 min, 98 to 95% A; 8.01 to 48 min, 95 to 75% A; 48 to 60 min, 75 to 60% A; 60 to 60.01 min, 60 to 10% A; 60.01 to 70 min, 10% A; 70 to 70.01 min, 10 to 98% A; 70.01 to 75 min, 98% A. Solvent A was acetonitrile (ACN)-H2O (v/v, 2:98, pH titrated to 10 by ammonium hydroxide), while solvent B was ACN-H2O (v/v, 90:10, pH titrated to 10 by ammonium hydroxide). Peptides from min 0 to 8 were purified by C18 chromatographic columns. Eluted fractions from min 8 to 60 were collected by an automated fraction collector and combined into 15 fractions.

A Q Exactive HF mass spectrometer (Thermo Fisher) equipped with a Nanospray Flex source (Thermo Fisher) was used for liquid chromatography-mass spectrometry (LC-MS/MS). Samples were loaded and separated by a C18 column (15 cm × 75 µm) on an EASY-nLCTM 1200 system (Thermo Fisher). A data-dependent acquisition mode was used for data collection. Full MS survey scans were acquired in the mass range of 350-1500 m/z with a mass resolution of 60000 and the automatic gain control (AGC) target value was set at 3×10^6^. The 20 most intense peaks in MS were fragmented with high-energy collisional dissociation (HCD) with collision energy of 32. MS/MS spectra were obtained with a resolution of 45000, an AGC target of 2×10^5^, a max injection time of 80 ms, an isolation window of 1.6m/z, and an intensity threshold of 5.0×10^6^. The Q Exactive HF dynamic exclusion was set for 30.0 s and run under positive mode.

### Protein identification

The acquired raw data were analysed with the ProteomeDiscoverer software (Thermo Fisher, version 2.4.1.15). The HCD MS/MS spectra were searched against the Uniprot-Homo sapiens database. Trypsin was set as the digestion enzyme with two allowed numbers of missed cleavage sites. Mass tolerances were set to 10 ppm and 0.02 Da for precursor and fragmentations. Alkylation of cysteine was considered as fixed modification. Carbamidomethylation of cysteines and TMT label on lysines and the N-terminus were set as static modifications. FDR was set to 0.01 and protein groups for quantification required at least one peptide. The protein concentrations were calculated by BCA method based on a standard curve and median normalization of proteomics data was performed. The abundance of the protein should be present in at least 3 samples in one patient group for subsequent analyses.

### Identification and annotation of protein with abundance

Putative differentially abundant proteins (DAPs) were delineated by the following criteria: fold change (FC) ≥ 1.50 or ≤ 0.67 and p-value < 0.05. The clustered heatmap of DAPs was generated by R Graphics. Correlation of DAPs was analysed by Pearson coefficients. The shared and unique DAPs of the three groups were visualized by a Venn diagram. Annotation of identified proteins was performed using Gene Ontology database (GO, http://www.blast2go.com/b2ghome; http://geneontology.org/) and Kyoto Encyclopedia of Genes and Genomes pathway (KEGG, http://www.genome.jp/kegg/).

### Enrichment analysis

GO and KEGG enrichment analysis were performed. A Benjamini-Hochberg-adjusted p-value of less than 0.05 was considered of statistical significance. Protein-protein interaction analysis was performed using STRING (https://string-db.org/). All proteins were ranked according to the degree of differential expression in Gene Set Enrichment Analysis (GSEA) using GSEA software 17. The top 10 terms based on enrichment scores were obtained from the biological process (BP), molecular function (MF) and cell component (CC) sub-ontology of GO analysis. The top 5 terms were visualized in ridge plots.

### Construction of protein-protein interaction network and module analysis

Protein-protein interaction (PPI) networks were constructed using STRING (https://string-db.org/). Significant modules in PPI networks and hub proteins (proteins that have higher possibility of being essential proteins 18) were visualized using MCODE (v2.0.2, degree cutoff 2, node score cutoff 0.2, maximum depth 100, K-core 2) with Cytoscape software (v3.9.1). Hub genes were obtained by intersecting the top 10 gene sets each sorted by Degree, Maximum Neighborhood Component (MNC), and Edge Percolated Component (EPC) algorithms using CytoHubba (v0.1) plug-in. ClueGO plugin (v.2.5.10) with Cytoscape was used to visualize the enriched pathways of hub genes.

### Muscle immunohistochemistry (IHC)

**Figure 1:**
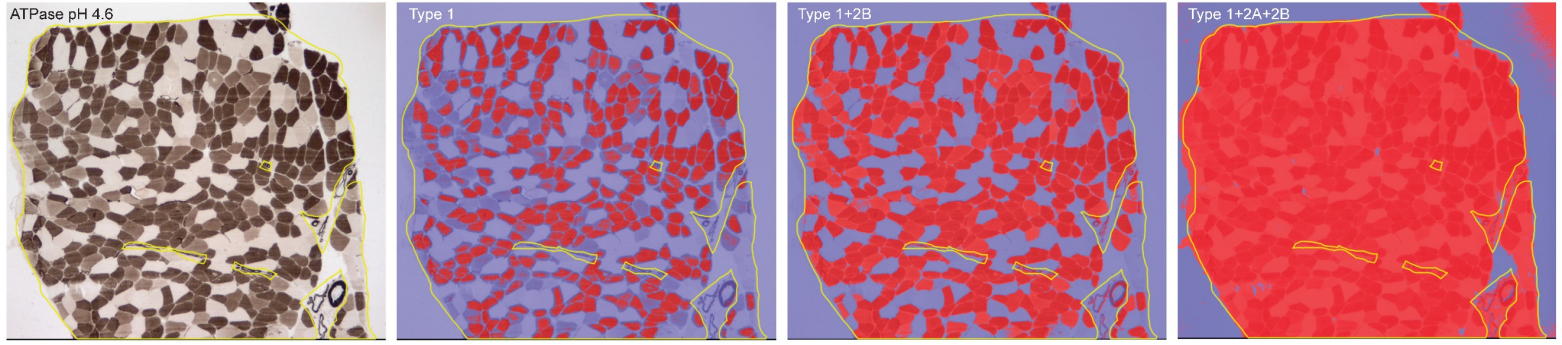
Illustration of ATPase (pH 4.6) staining of muscle section from a desminopathy patient, and the colour deconvolution by different threshold numbers that enables fiber type differentiation.

Sections of 8 µm thickness were cut using a cryostat (Leica CM1900). Hematoxylin/eosin (HE) and ATPase (pH 4.2, 4.6, 9.6) staining was performed. The degree of interstitial tissue proliferation of muscle tissues was determined as: no, mild, moderate and severe proliferation by two independent observers (Q.L. and Y.B.L.). Photomicrographs of ATPase pH 4.6 were taken in one to three fields under 40× magnification depending on the size of the muscle sections. The QuPath software (v.0.4.4) was used for quantification of the area of interest (AOI). During colour deconvolution, different threshold numbers were applied to differentiate type 1, 2A and 2B muscle fibers. The AOI for type 1 fibers was defined as the percentage of the immunoreactive area to the whole muscle section area of interest with the highest threshold number (ranging from 0.3 to 0.42). The AOI for type 2B fibers was defined as the area with the intermediate threshold number (0.18 to 0.28) minus the AOI of type 1 fibers. The AOI for type 2A fibers was defined as the area with the lowest threshold number (0.01 to 0.05) minus the AOI of the area with the intermediate threshold number (Fig. 1).

### Statistical analysis

The significant difference of protein abundance was analyzed with independent t-test between two groups. For GO/KEGG analysis, the species proteins were used as the background list and the DAP list as the candidate list selected from the background list. The hypergeometric distribution testing was used to calculate the p-value for whether the functional sets were significantly enriched in the DAP list. Then the Benjamini-Hochberg procedure was utilized to obtain FDR values. Analysis regarding muscle IHC was performed using SPSS (IBM, version 29.0). The comparison of the distribution of fiber type area was evaluated using the Shapiro-Wilk test, and the variance was assessed with the Levene's test. Two-sided independent t-tests were used to compare the AOI between groups.

## Results

### Identification of DAPs

**Figure 2:**
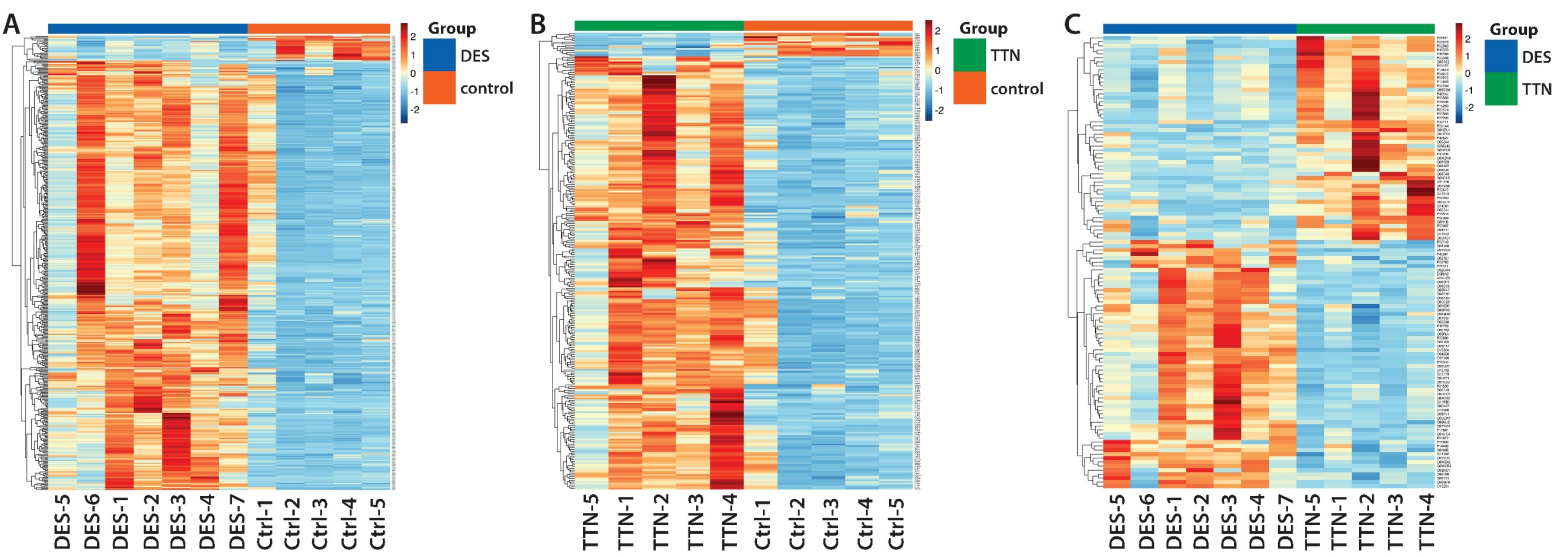
Heatmaps visualizing hierarchical clustering of DAPs.

Using an FDR value < 0.01, 32949 peptides and 3591 proteins were identified as belonging to the muscle proteome in this study. A total of 3296 proteins with abundance were used for subsequent analyses. The hierarchical clustering of DAPs was visualized by heatmaps (Fig. 2).

**Figure 3:**
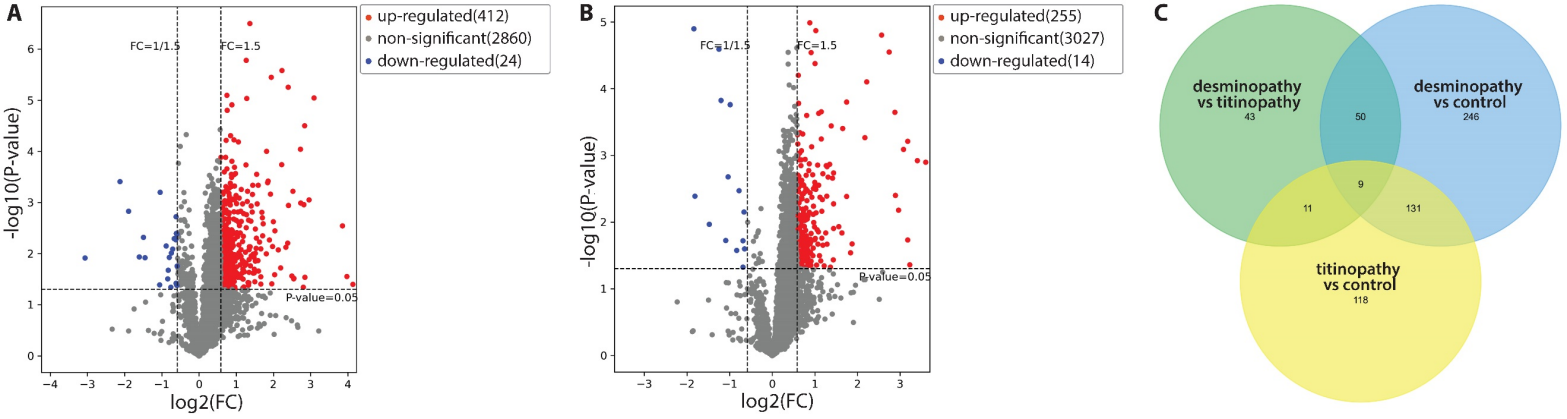
Volcano plots demonstrating the DAPs between the desminopathy and control groups (A), and between the titinopathy and control groups (B). C. a Venn diagram showing overlapping and distinct DAPs among the three groups.

Compared with control samples, there were 436 DAPs in the desminopathy group (412 upregulated and 24 downregulated), and 269 DAPs in the titinopathy group (255 upregulated and 14 downregulated, Fig. 3A, B). When comparing the desminopathy with the titinopathy group, there were fewer DAPs (62 upregulated and 51 downregulated). The number of overlapping and non-overlapping DAPs were visualized in a Venn diagram (Fig. 3C). Table 1 summarized the most dysregulated DAPs in these groups. The identified proteins and all DAPs were included in Supplementary Table 2-6.

### Annotation of desminopathy-related DAPs and pathway enrichment analysis

GO analysis found that the DAPs in desminopathy were enriched in the cytosol, membrane, extracellular exosome in the 'cellular component' sub-ontology; in protein localization to plasma membrane, glycolytic process in the 'biological process' sub-ontology; and in actin binding, actin filament binding, structural constituent of cytoskeleton in the 'molecular function' sub-ontology (Supplementary Fig. 1A). Despite its capability of identifying dysregulated pathways, GO analysis was unable to reveal the overall functional state of each pathway. We therefore further performed GSEA on the three sub-ontologies to investigate the directionality of the enriched terms. Biological processes and pathways mainly involved in mitochondrial energy production, muscle contraction and cytoskeleton organization were significantly suppressed (Fig. 5A, D, Supplementary Table 2). Activated cellular components and pathways were mostly related to the extracellular matrix (ECM), such as integrin binding, extracellular space, and collagen fibril organization (Fig. 5G, J, Supplementary Table 2). KEGG enrichment pathway analysis revealed similar enriched pathways (Supplementary Fig. 1B).

MCODE delineated 18 clusters of interconnecting sub-networks. Hub genes identified by CytoHubba were *ACTB*, *CDC42*, *CAT*, *ANXA2*, *ITGB1* and *CD44* (Table 2). These genes were not enriched in any particular pathways.

### Annotation of titinopathy-related DAPs and pathway enrichment analysis

The DAPs in titinopathy compared with control samples were also enriched in cellular component as in the cytosol, cytoplasm, extracellular exosome and focal adhesion (Supplementary Fig. 1C, 1D). GSEA identified overall downregulated mitochondrial-related pathways such as the tricarboxylic acid cycle, mitochondrial electron transport, and NADH dehydrogenase activity, which was similar to desminopathy (Supplementary Table 7).

Another markedly suppressed functional group was ECM, including ECM organization and ECM structural constituent (Fig. 5B, E, Supplementary Table 7). However, the gluconeogenesis pathway in titinopathy was upregulated (NES 1.535, p-value 0.026), and the glycolytic process was slightly upregulated without statistical significance (NES 1.020, p-value 0.466). Other upregulated terms included microtubule associated complex, negative regulation of cell migration and endocytic recycling. Twelve sub-networks and nine hub genes were identified (Fig. 6A, Table 2).

### Comparison between desminopathy and titinopathy

**Figure 4:**
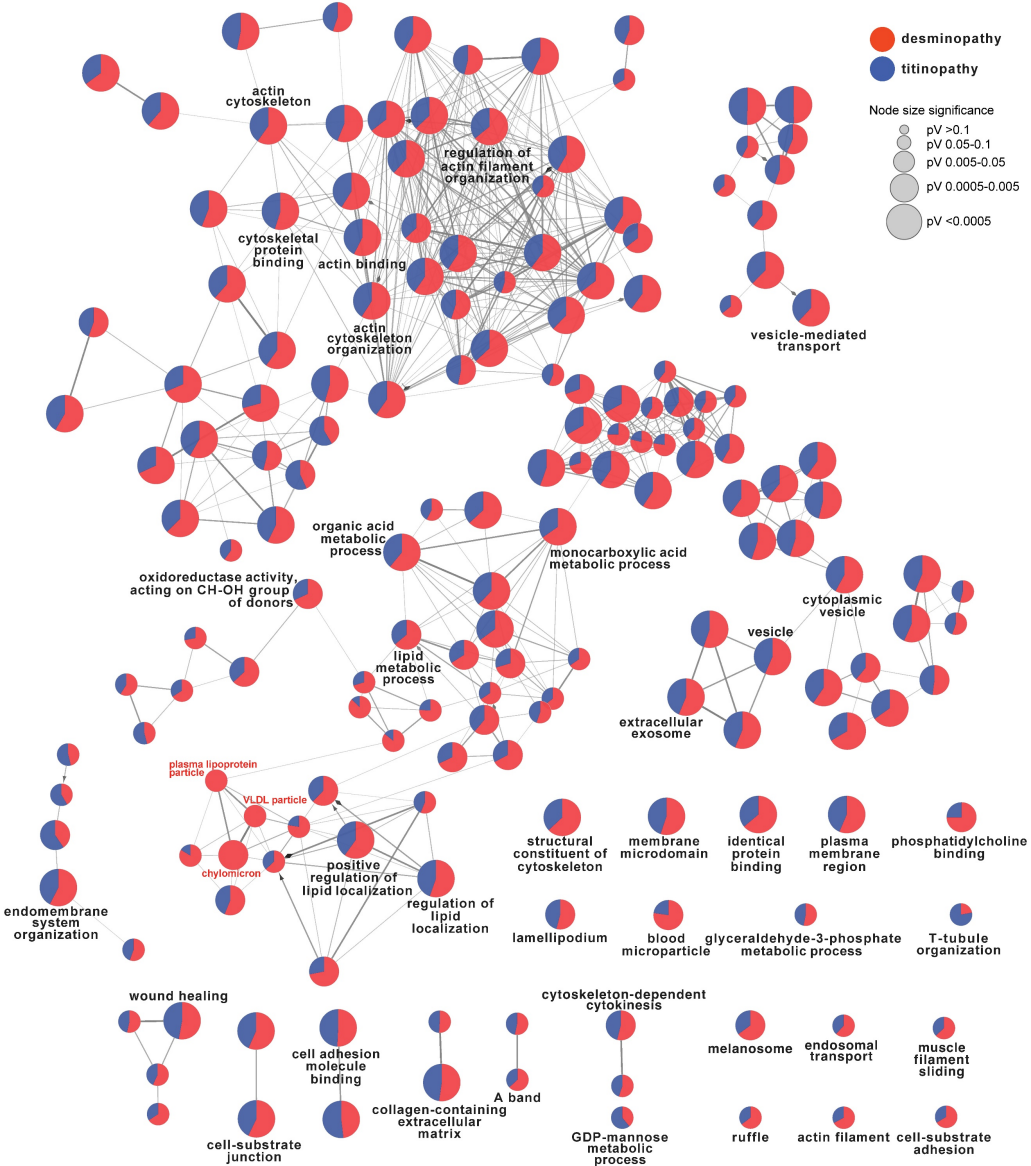
ClueGO comparison of desminopathy- and titinopathy-DAPs. Only terms at upper levels (more generalized terms, in black font) and the uniquely enriched pathways (in red font) were shown.

To identify the common and different pathways between desminopathy and titinopathy, we first used ClueGO to compare the two sets of DAPs in desminopathy and titinopathy. Most enriched pathways were shared between these two phenotypes except for lipid metabolism, which was dysregulated in only desminopathy (Fig. 4). The common hub genes in desminopathy and titinopathy were cytoskeleton- (*ACTB*, *ANXA2*), and ECM-related (*CD44*).

**Figure 5:**
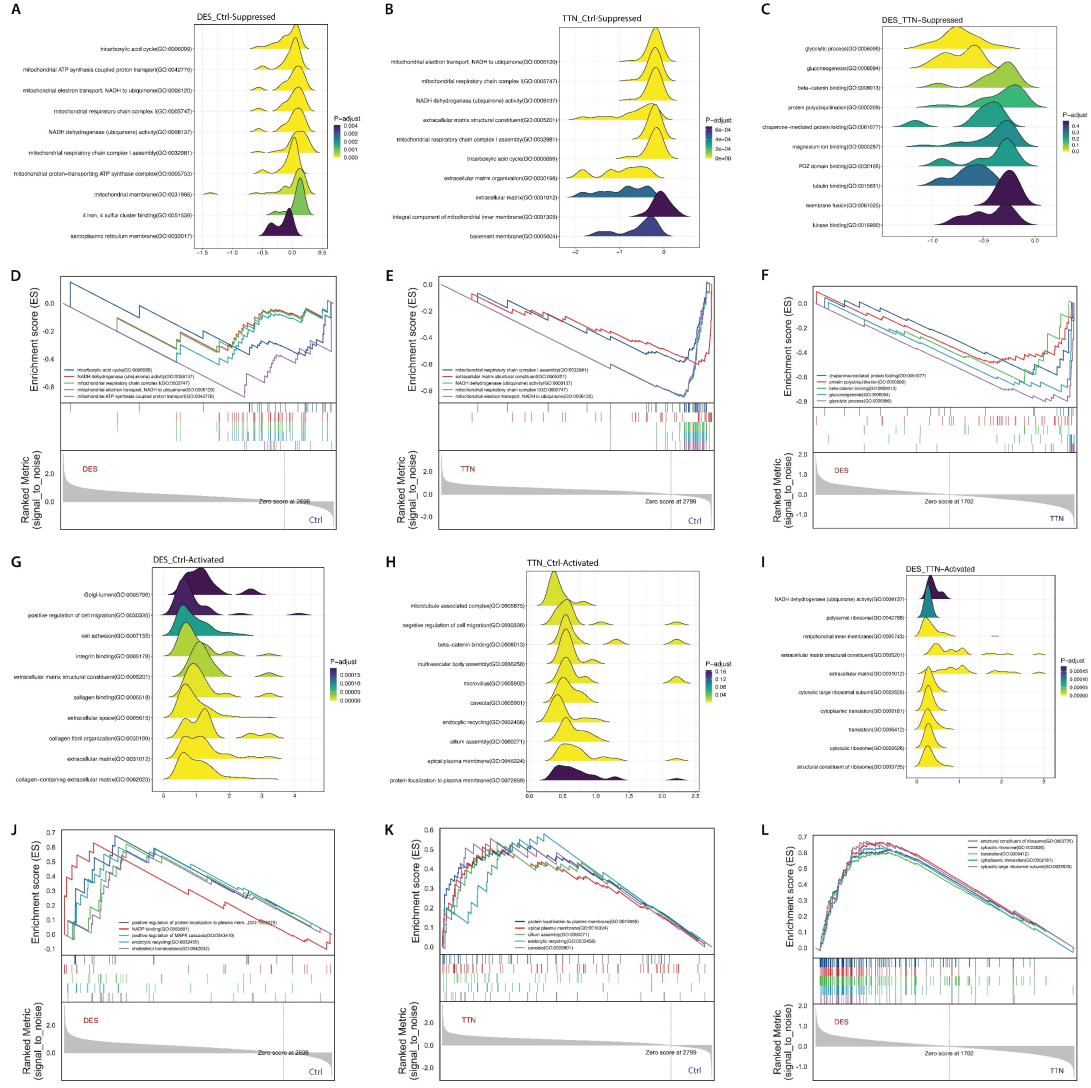
GSEA results using GO terms. Ridge plots showing the top 10 dysregulated pathways in desminopathy (A, downregulated; G, upregulated), in titinopathy (B, downregulated; H, upregulated), and in desminopathy compared with titinopathy (C, downregulated; I, upregulated). GSEA plots showing the top 5 dysregulated pathways in desminopathy (D, downregulated; J, upregulated), in titinopathy (E, downregulated; K, upregulated), and in desminopathy compared with titinopathy (F, downregulated; L, upregulated)

**Figure 6:**
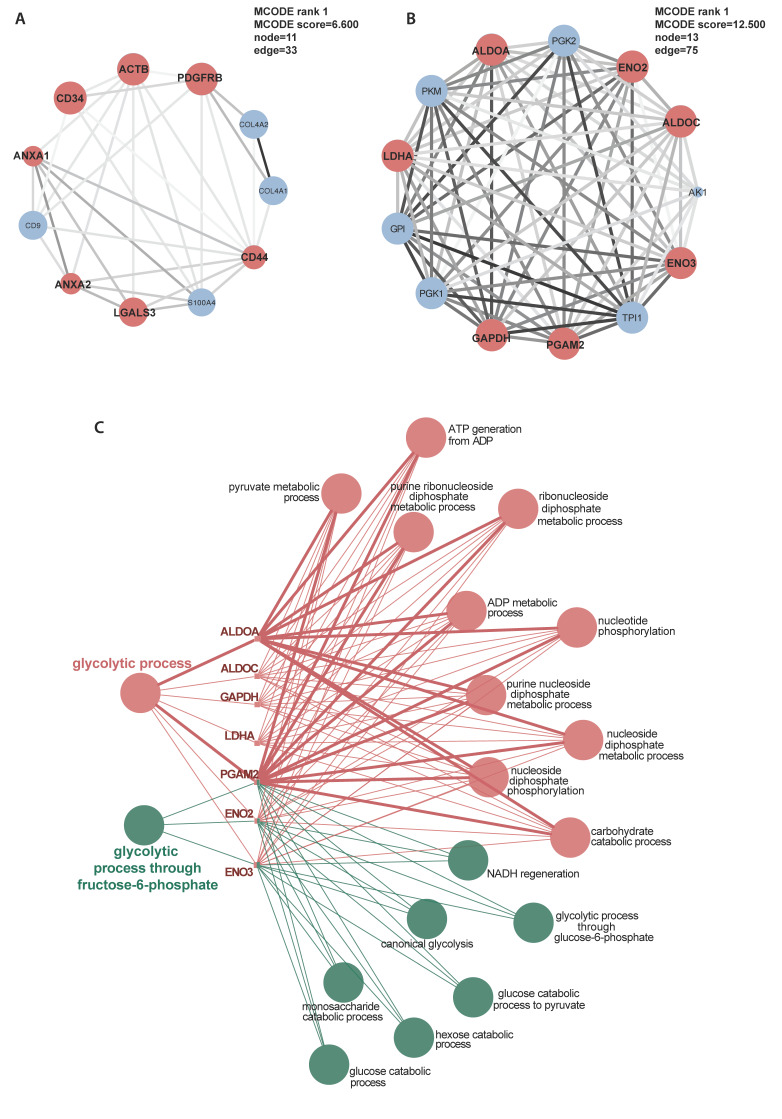
Selected illustration of sub-networks of the DAPs in titinopathy compared with controls (A) and in desminopathy compared with titinopathy (B). Hub genes were designated as red coloured nodes. Sizes of nodes increase with computed scores. Degrees of shade of connecting lines also positively correlate with gene co-expression. C, enriched pathways of the hub genes in desminopathy compared with titinopathy.

We next used the DAPs generated by directly comparing the two MFM subtypes for GSEA (Supplementary Table 7). Compared with titinopathy, glycolytic process and gluconeogenesis were the most significantly downregulated biological processes in desminopathy (Fig. 5C, F, Supplementary Fig. 1E, F). Upregulated pathways in desminopathy were ribosome, translation and ECM (Fig. 5I, L). Mitochondria-related pathways and processes were less suppressed in desminopathy than in titinopathy. Interestingly, all seven hub genes were grouped into cluster 1 as identified by MCODE (Fig. 6B), and were involved in the glycolytic process (Fig. 6C, Table 2). Visualized expression levels of all hub genes were presented in Supplementary Fig.2.

### Histochemistry findings of desminopathy and titinopathy

**Figure 7:**
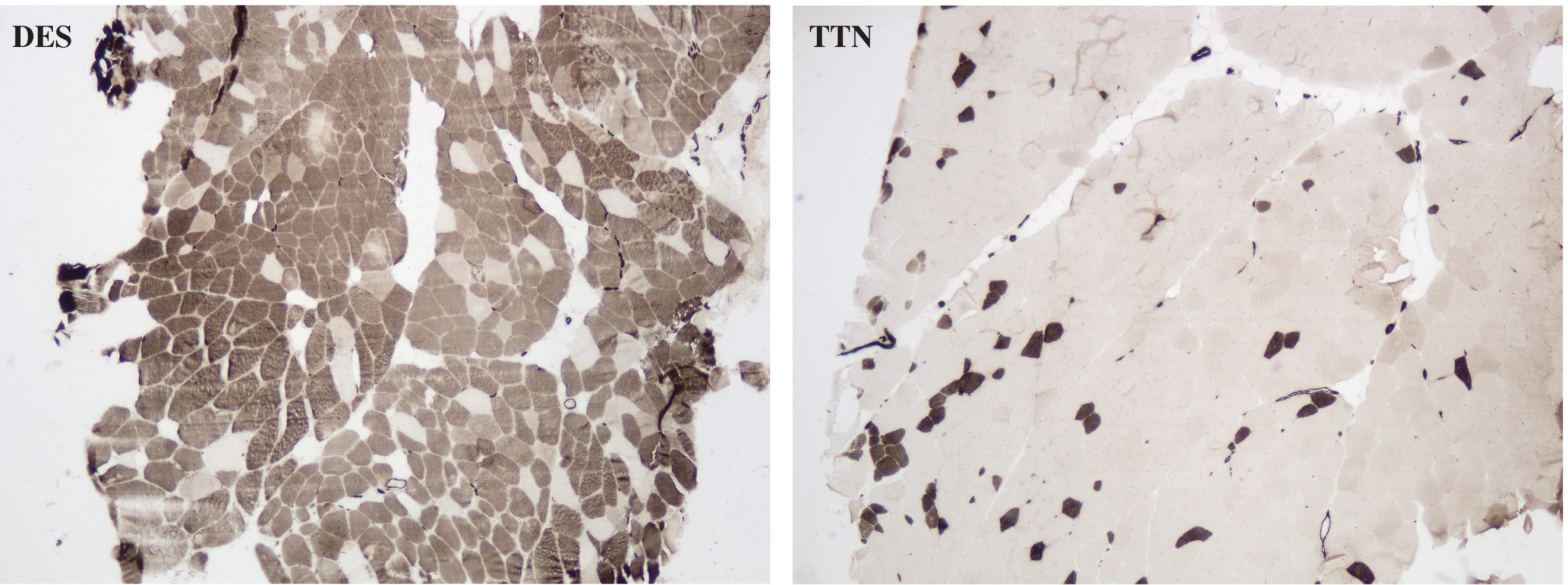
Photomicrophaphs of ATPase (pH 4.6) staining showing type 1 predominance in a desminopathy patient (DES) and type 2 predominance in a titinopathy patient (TTN). ×40 magnification.

There was no difference in the degree of interstitial tissue proliferation between desminopathy and titinopathy. Desminopathy had a higher proportion of type 1 fibers compared to titinopathy (p = 0.02, Fig. 7), but the differences in type 2A and 2B were not significant (p = 0.076, 0.709 respectively, Fig. 7).

## Discussion

The present proteomic study has identified common mitochondrial abnormalities, particularly complex I dysfunction, in both desminopathy and titinopathy. The two MFM subtypes also have distinct pathogenic processes. Desminopathy has more severe cytoskeleton disorganization and activated ECM-related process. Disturbed cholesterol metabolism is only found in desminopathy. On the other hand, titinopathy shows significant ECM suppression. Glucose metabolism is suppressed in desminopathy, while activated in titinopathy.

Our study has revealed pronounced dysregulation of cytoskeleton in both desminopathy and titinopathy. Myofiber cytoskeleton consists of three interconnecting networks: microtubules, actin filaments (F-actin) and desmin intermediate filaments (IFs). Microtubules play a major role in the subcellular positioning of organelles, especially myonuclei 19. F-actin participates in muscle contraction by associating with myosin, and cell motility through dynamic re-organization 20. IFs provide structural support to cells. All three cytoskeleton sub-networks are upregulated in desminopathy and titinopathy, with the actin cytoskeleton being the most overexpressed in desminopathy and the microtubule in titinopathy. Desmin is a muscle-specific type III IF protein that is located at the periphery of the Z disc and connects adjacent sarcomeres. It builds a scaffold structure around Z discs, thus ensures lateral force transmission in the form of costamere. Mutant desmin is found to destabilize F-actin by its defective interaction with nebulin and the malfunctioning desmin dimer complex 7, 21. Malfunction of the actin cytoskeleton compromises myofiber integrity, signal and force transduction and cell migration. Compared with desmin, which contributes to the lateral compliance of myofibers, the gigantic protein titin plays a more significant role in regulating sarcomere longitudinal force and muscle passive tension through its association with both thin and thick filaments. It spans across the Z disc and M line, and functions as a spring for myofibril contraction and stretching. Titin is connected to microtubules through the muscle-specific RING finger proteins (MURFs). Microtubules transiently assists in the elongation of titin during sarcomere assembly 22. However, how defective titin affects microtubules remains unclear.

Glucose metabolism, including glycolysis, gluconeogenesis, and PPP, shows different functional state in desminopathy and titinopathy. The expression of five enzymes related to glycolysis and glyconeogenesis are suppressed in desminopathy, suggesting compromised glucose metabolism. Interestingly, these enzymes are involved in three consecutive catalytic reactions, involving the conversion from 1,3-bisphosphoglycerate to phosphoenolpyruvate. Our finding contradicts Elsnicova's study, which used desmin null cardiomyocytes and demonstrated increased glucose metabolism 13. The mutant desmin protein in our patients is either truncated or contains an amino acid substitution. Another difference between our and Elsnicova's study is that the expression of the mutant desmin is elevated, unlike the complete absence of desmin expression in the previous study. This offers one possible explanation for the discrepancy. In a rat model for titinopathy, the metabolism of heart was found to shift from fatty acid to glycolysis 23, which is in accordance with our findings. We have identified six glucometabolism regulatory genes that are differentially abundant in MFM. Products of all these genes (fructose-biphosphate aldolase, gylceraldehyde-3-phosphate dehydrogenase, phosphoglycerate mutase, enolase and lactate dehydrogenase) are involved in glycolysis process, suggesting metabolism shift in MFM.

The unbalanced glycolytic process in desminopathy and titinopathy can be at least partly explained by fiber type disproportion as illustrated by our ATPase staining. The type 1 fibers were increased and type 2 fibers were accordingly decreased in desminopathy compared with titinopathy muscles. In muscle tissues, type 1 fibers are oxidative fibers that depend on mitochondrial aerobic respiration to generate ATP. Type 2B fibers are fast glycolytic fibers that mainly use anaerobic glycolysis for energy production, whereas type 2A fibers are intermediate fibers whose energy machinery involves both oxidative phosphorylation and glycolysis. In agreement with the ATPase staining results, the type 2 fiber-associated proteins alpha-actin-3 (*ACTN3*) and troponin C (*TNNC*) are among the most downregulated DAPs in desminopathy compared to titinopathy (Table 1). The mechanism underlying muscle fiber type switching warrants further investigation.

The difference in ECM identified by the present study cannot be explained by disease duration as our desminopathy patients have shorter duration than the titinopathy patients do (7.33±2.34 yr vs 11.83±10.99 yr). Neither is this disparity observable on muscle pathology, as we did not find higher extent of fibrosis in desminopathy muscles. Apart from being a pivotal member of costamere, desmin also associates with transmembrane receptor integrins, which control cell adhesion and migration by binding to components of ECM 24. The heart of desmin null mice demonstrated increased levels of transforming growth factor-1 and extensive fibrosis 25. The upregulated cell migration, adhesion process, and ECM found in our desminopathy patients is likely to be a compensatory mechanism of the defective desmin. Dynamic ECM remodeling is also involved in muscle repair. The ECM is partially degraded to facilitate the migration of activated satellite cells to the site of the damaged muscle 26. Fibroblasts are activated to transiently produce ECM components including type I and III collagen, laminin and proteoglycan during muscle regeneration. The activated ECM related processes in desminopathy may also be related to active muscle regneration. Titinopathy, on the other hand, has reduced expression of ECM component proteins, including type I and IV collagen, and fibrillin-1. An interesting finding from Hettige et al.'s study on a titinopathy mouse model (muscular dystrophy with myositis, mdm) is that the genes related to ECM are downregulated in the extensor digitorum longus, but upregulated in the psoas and soleus 27, reflecting differential reactions to *TTN* mutations from different muscle groups. The majority of muscles in this study were taken from the biceps brachii, except for two desminopathy cases (DES-6 and -7), which were from the gastrocnemius. It is not clear if the differential trend of ECM is replicated in human patients.

Desmin participates in the maintenance of mitochondrial localization, shape and respiratory chain function 28. It anchors mitochondria to sarcomeres. This spatial proximity facilitates timely intake of ATP once generated by the respiratory chain. Desminopathy muscles exhibit altered mitochondrial morphology and function. Mutant desmin reduces protein levels of all five respiratory chain complexes and suppresses respiratory function, especially complex I activity 16, 29. A weighted gene co-expression network analysis using RNA-Seq data from the muscles of mdm mice also found reduced complex I activity 27, which is in accordance with our findings.

The extracellular exosome is found to be significantly dysregulated in both desminopathy and titinopathy. Exosomes have rarely been studied in MFM. One study found that the structure and function of mitochondria were compromised in desminopathy, causing subsequent mitochondrial DNA (mtDNA) release in form of exosome in late stage of myofiber differentiation 29. Exosomes secreted by myotubes have been shown to direct stem cells to myogenesis 30. HyperCKemia as well as active muscle necrosis and regeneration is common in MFM. It is likely that exosomes, at least partly containing mtDNA, play an important part in inducing muscle regeneration in MFM.

The present study has limitations. Due to the rarity of MFM, the sample sizes of desminopathy and titinopathy are small. For the delineation of putative DAPs, we have employed the p-values instead of adjusted p-values obtained through multiple testing corrections. This method leverages a more permissive inclusion of potential DAPs in exchange for the number of false positives 31. The use of multiple testing corrections in proteomic studies may be limited by several factors such as medium scale, ratio compression in labelled experiments and the possibility of increased false negatives, as extensively discussed in 32. Therefore, we also included the FC values for DAP definition. This is an exploratory study in which we aim to identify the concordant differences in pathways in MFM muscles using the potential DAP repertoire. We have refrained from drawing conclusions on any single DAP identified by this method.

The salient point of the present study is that MFM patients of different subtypes share cytoskeleton dys-organization and mitochondrial dysfunction, albeit to varying degrees. One important difference in the pathomechanisms of desminopathy and titinopathy is the differentially dysregulated glycolytic process and ECM. Fiber type disproportion at least partly accounts for the suppressed glycolytic process in desminopathy. This offers future direction for targeted therapy for MFM.

## Supplementary Material

Supplementary figures, table legends.

Supplementary tables.

## Figures and Tables

**Table 1:** Top DAPs

	desminopathy vs control				titinopathy vs control				desminopathy vs titinopathy			
	Gene Name	P-value	log2(FC)	FC	Gene Name	P-value	log2(FC)	FC	Gene Name	P-value	log2(FC)	FC
Down	*ACTN3*	0.012191	-3.06825	0.119224	*COL4A2*	1.26E-05	-1.83598	0.280102	*ACTN3*	0.016517	-2.09967	0.233312
	*ATP1A3*	0.000392	-2.12692	0.228946	*ATP1A3*	0.004081	-1.8137	0.28446	*GSTT1*	0.000965	-1.72241	0.303042
	*AQP4*	0.001474	-1.89799	0.268316	*FBN1*	0.010756	-1.47691	0.359256	*AQP4*	0.008475	-1.39373	0.38058
	*MYH1*	0.01166	-1.60529	0.328669	*MYO3B*	2.56E-05	-1.2532	0.419515	*CHORDC1*	0.009503	-1.16802	0.445032
	*CDK12*	0.004814	-1.4954	0.354683	*COL4A1*	0.00015	-1.20091	0.434999	*TNNC2*	0.000385	-1.12318	0.459082
	*MYH4*	0.012062	-1.4498	0.366071	*IDI2*	0.018773	-1.09153	0.469264	*ENO2*	0.002649	-1.07946	0.473205
	*ANKRD6*	0.040654	-1.0602	0.479565	*FNDC3A*	0.002096	-1.0381	0.486968	*PGAM2*	0.013859	-1.00695	0.497597
	*RAP1GDS1*	0.000637	-1.04973	0.483059	*TTN*	0.000174	-0.9861	0.504839	*MTPN*	0.005946	-0.92514	0.526629
	*ENO2*	0.007077	-0.88716	0.540679	*COL1A1*	0.026492	-0.83717	0.559741	*DOCK4*	0.049735	-0.90933	0.532431
	*MYLK2*	0.031148	-0.84002	0.558637	*SDHC*	0.003397	-0.77786	0.58323	*GPI*	3.37E-06	-0.90147	0.535341
	*PDLIM7*	0.021057	-0.82767	0.563439	*ZNF568*	0.047485	-0.68983	0.619927	*TMA7*	0.006315	-0.89773	0.536732
	*PGAM2*	0.011868	-0.80222	0.573465	*DUSP26*	0.018944	-0.68877	0.620381	*PGK1*	0.023514	-0.85683	0.552165
	*TNNC2*	0.045652	-0.75791	0.591354	*FAM91A1*	0.007037	-0.66128	0.632318	*UBE2G1*	0.013461	-0.82623	0.564002
	*PGK1*	0.009721	-0.74737	0.595687	*RAP1GDS1*	0.025243	-0.65104	0.636821	*PGK2*	0.016095	-0.81473	0.568515
	*ENO3*	0.008165	-0.70967	0.611458					*CYFIP1*	0.007538	-0.80936	0.570636
Up	*MAP4K4*	0.039986	4.132363	17.5374	*ALDOB*	0.001259	3.597461	12.10441	*DDN*	0.003701	3.235111	9.415981
	*OSGEPL1*	0.027986	3.96956	15.66595	*CPQ*	0.001193	3.400836	10.56218	*MLF2*	0.001815	2.300981	4.927926
	*DDN*	0.002846	3.850608	14.42609	*MAP4K4*	0.043816	3.226091	9.35729	*TRMT61A*	0.002633	1.936327	3.827301
	*ALDOB*	9.03E-06	3.08675	8.495798	*CALB1*	0.000612	3.174984	9.031616	*MARCHF7*	0.002466	1.870884	3.657566
	*MLF2*	0.000892	2.956061	7.760023	*OSGEPL1*	0.018554	3.173536	9.022556	*MLF1*	0.003271	1.795941	3.472419
	*DNAH3*	0.028961	2.84415	7.180828	*FBP1*	0.000812	3.0751	8.427473	*DNAJB6*	0.000524	1.739207	3.338515
	*CPQ*	3.14E-05	2.838331	7.151922	*ADH6*	0.006588	2.95893	7.77547	*ZNF568*	0.019101	1.691008	3.228823
	*CALB1*	0.001104	2.818311	7.053362	*SLC5A2*	0.003975	2.885472	7.389474	*RTL8B*	0.039936	1.583283	2.99651
	*ARFIP1*	0.045415	2.798449	6.956922	*FMO1*	0.000226	2.876528	7.343808	*NECAB3*	0.022724	1.530698	2.889256
	*TRMT61A*	0.001025	2.728896	6.62948	*GSTT1*	2.81E-05	2.737856	6.670784	*DUSP26*	0.001219	1.45479	2.741166
	*FBP1*	9.05E-05	2.724798	6.610675	*ALDH8A1*	1.58E-05	2.562128	5.90578	*SNRNP35*	0.026466	1.282464	2.432541
	*SOWAHD*	0.030559	2.551236	5.86136	*SLC9A3R1*	7.92E-05	2.216567	4.64786	*PPP1R3C*	0.036009	1.263838	2.401337
	*RCBTB2*	0.031026	2.54483	5.835395	*AASS*	0.00054	2.173315	4.510585	*MAGED1*	0.000785	1.23865	2.359776
	*ADH6*	0.000605	2.521868	5.743253	*SOWAHD*	0.021241	1.873377	3.663891	DDIT4L	0.01177	1.214473	2.320561
	*NQO1*	0.027547	2.490768	5.62077	*ORC1*	0.028724	1.836466	3.571342	SSBP2	0.020458	1.200125	2.297596

FC: fold change

**Table 2:** Hub genes

Gene name	Protein name	log2(FC)	FC	P-value
**Desminopathy versus control**				
*ANXA2*	Annexin A2	1.33023	2.51443	0.00220
*ACTB*	Actin, cytoplasmic 1	1.16488	2.24215	0.00106
*CDC42*	Cell division control protein 42 homolog	0.78513	1.72324	0.02153
*CD44*	CD44 antigen	0.74173	1.67218	0.01505
*CAT*	Catalase	0.71938	1.64648	0.00633
*ITGB1*	Integrin beta-1	0.63291	1.55069	0.04343
*ENO2*	Gamma-enolase	-0.88716	0.54068	0.00708
**Titinopathy versus control**				
*ACTB*	Actin, cytoplasmic 1	1.14674	2.21413	0.00057
*ANXA2*	Annexin A2	0.96944	1.95808	0.01012
*LGALS3*	Galectin-3	0.95451	1.93792	0.03182
*ANXA1*	Annexin A1	0.83889	1.78868	0.02650
*TAGLN*	Transgelin	0.78088	1.71818	0.00877
*CD34*	Hematopoietic progenitor cell antigen CD34	0.73783	1.66766	0.00282
*PDGFRB*	Platelet-derived growth factor receptor beta	0.60825	1.52441	0.01115
*CD44*	CD44 antigen	0.59959	1.51528	0.01268
*COL1A1*	Collagen alpha-1(I) chain	-0.83717	0.55974	0.02649
**Desminopathy versus titinopathy**				
*ENO2*	Gamma-enolase	-1.07946	0.47320	0.00265
*PGAM2*	Phosphoglycerate mutase 2	-1.00695	0.49760	0.01386
*ENO3*	Beta-enolase	-0.78998	0.57835	0.00563
*GAPDH*	Glyceraldehyde-3-phosphate dehydrogenase	-0.78044	0.58219	0.00671
*LDHA*	L-lactate dehydrogenase A chain	-0.76727	0.58753	0.01314
*ALDOA*	Fructose-bisphosphate aldolase A	-0.69984	0.61564	0.00220
*ALDOC*	Fructose-bisphosphate aldolase C	-0.65918	0.63324	0.00570

FC: fold change
