# Supplementary material for: Common and Key Differential Pathogenic Pathways in Desminopathy and Titinopathy
Source: Int J Med Sci. 2024 Aug 1;21(11):2040–51. doi: 10.7150/ijms.97797 (PMC11373559; doi:10.7150/ijms.97797)
Supplement: Supplementary file 1 — Supplementary figures, table legends. [file ijmsv21p2040s1.pdf]

Supplementary materials

Supplementary Figure 1 Bubble plots of GO and KEGG analysis results.

Supplementary Figure 2 Expression levels of hub genes in the three groups

Supplementary Table 1 Demographics of MFM patients

Supplementary Table 2 Identified protein

Supplementary Table 3 Proteins used for subsequent analysis

Supplementary Table 4 Putative DAPs in desminopathy compared with titinopathy

Supplementary Table 5 Putative DAPs in desminopathy compared with controls

Supplementary Table 6 Putative DAPs in titinopathy compared with controls

Supplementary Table 7 Most dysregulated pathways by GSEA

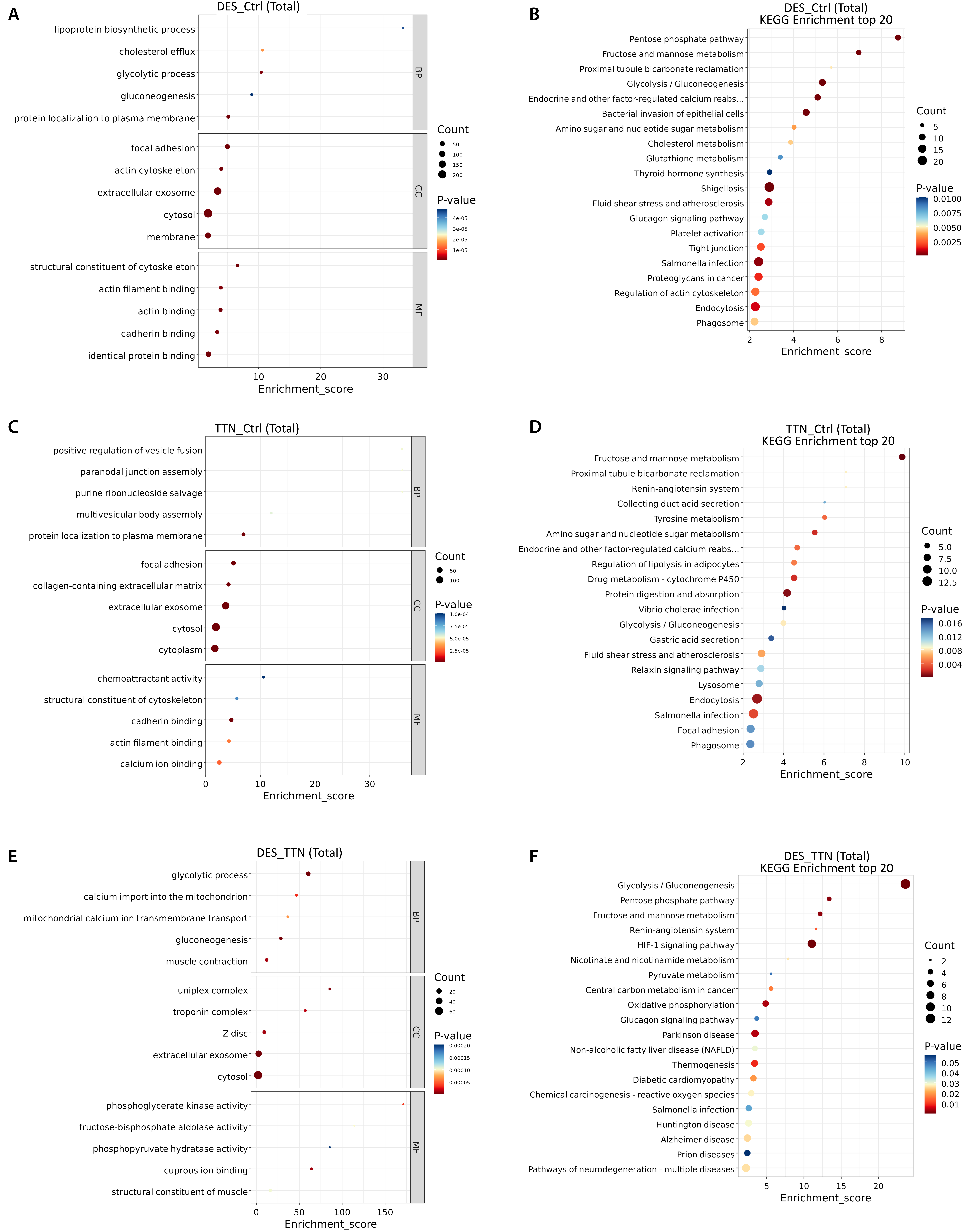
A
DES_Ctrl (Total)
lipoprotein biosynthetic process
cholesterol efflux
glycolytic process
gluconeogenesis
protein localization to plasma membrane
focal adhesion
actin cytoskeleton
extracellular exosome
cytosol
membrane
structural constituent of cytoskeleton
actin filament binding
actin binding
cadherin binding
identical protein binding
BP
CC
MF
Count
P-value
Enrichment_score
B
DES_Ctrl (Total)
KEGG Enrichment top 20
Pentose phosphate pathway
Fructose and mannose metabolism
Proximal tubule bicarbonate reclamation
Glycolysis / Gluconeogenesis
Endocrine and other factor-regulated calcium reabs...
Bacterial invasion of epithelial cells
Amino sugar and nucleotide sugar metabolism
Cholesterol metabolism
Glutathione metabolism
Thyroid hormone synthesis
Shigellosis
Fluid shear stress and atherosclerosis
Glucagon signaling pathway
Platelet activation
Tight junction
Salmonella infection
Proteoglycans in cancer
Regulation of actin cytoskeleton
Endocytosis
Phagosome
Count
P-value
Enrichment_score
C
TTN_Ctrl (Total)
positive regulation of vesicle fusion
paranodal junction assembly
purine ribonucleoside salvage
multivesicular body assembly
protein localization to plasma membrane
focal adhesion
collagen-containing extracellular matrix
extracellular exosome
cytosol
cytoplasm
chemoattractant activity
structural constituent of cytoskeleton
cadherin binding
actin filament binding
calcium ion binding
Count
P-value
Enrichment_score
D
TTN_Ctrl (Total)
KEGG Enrichment top 20
Fructose and mannose metabolism
Proximal tubule bicarbonate reclamation
Renin-angiotensin system
Collecting duct acid secretion
Tyrosine metabolism
Amino sugar and nucleotide sugar metabolism
Endocrine and other factor-regulated calcium reabs...
Regulation of lipolysis in adipocytes
Drug metabolism - cytochrome P450
Protein digestion and absorption
Vibrio cholerae infection
Glycolysis / Gluconeogenesis
Gastric acid secretion
Fluid shear stress and atherosclerosis
Relaxin signaling pathway
Lysosome
Endocytosis
Salmonella infection
Focal adhesion
Phagosome
Count
P-value
Enrichment_score
E
DES_TTN (Total)
glycolytic process
calcium import into the mitochondrion
mitochondrial calcium ion transmembrane transport
gluconeogenesis
muscle contraction
uniplex complex
troponin complex
Z disc
extracellular exosome
cytosol
phosphoglycerate kinase activity
fructose-bisphosphate aldolase activity
phosphopyruvate hydratase activity
cuprous ion binding
structural constituent of muscle
Count
P-value
Enrichment_score
F
DES_TTN (Total)
KEGG Enrichment top 20
Glycolysis / Gluconeogenesis
Pentose phosphate pathway
Fructose and mannose metabolism
Renin-angiotensin system
HIF-1 signaling pathway
Nicotinate and nicotinamide metabolism
Pyruvate metabolism
Central carbon metabolism in cancer
Oxidative phosphorylation
Glucagon signaling pathway
Parkinson disease
Non-alcoholic fatty liver disease (NAFLD)
Thermogenesis
Diabetic cardiomyopathy
Chemical carcinogenesis - reactive oxygen species
Salmonella infection
Huntington disease
Alzheimer disease
Prion diseases
Pathways of neurodegeneration - multiple diseases
Count
P-value
Enrichment_score

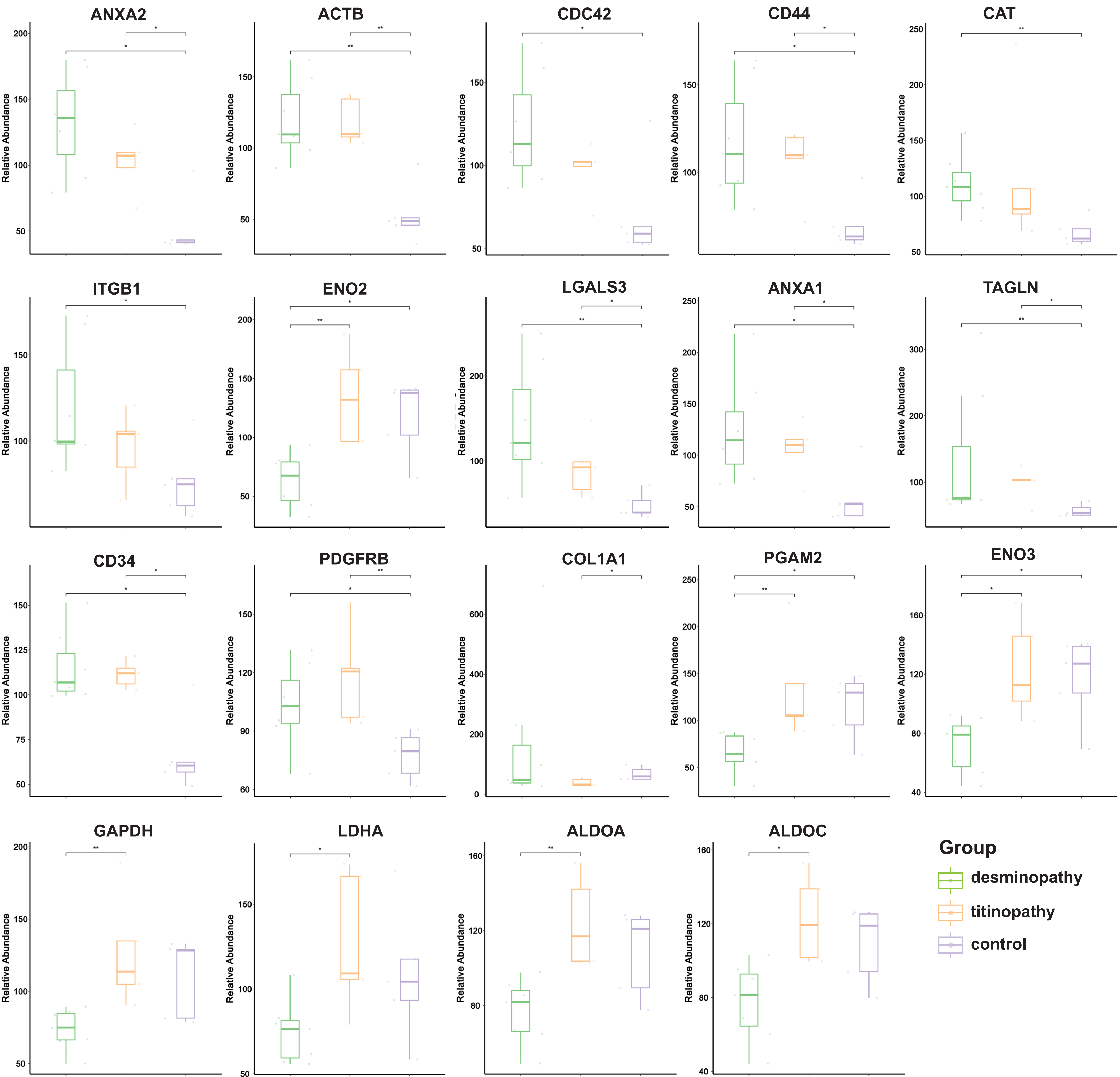
ANXA2
ACTB
CDC42
CD44
CAT
ITGB1
ENO2
LGALS3
ANXA1
TAGLN
CD34
PDGFRB
COL1A1
PGAM2
ENO3
GAPDH
LDHA
ALDOA
ALDOC
Relative Abundance
Group
desminopathy
titinopathy
control
